# RETRACTION: Efficacy of Stem Cell Therapy for Diabetic Foot: Clinical Evidence From Meta‐Analyses

**DOI:** 10.1111/iwj.70580

**Published:** 2025-04-18

## Abstract

**RETRACTION:** ZhangY. , LiJ. , and LiuM. , “Efficacy of Stem Cell Therapy for Diabetic Foot: Clinical Evidence From Meta‐Analyses,” International Wound Journal 21, no. 4 (2024): e14632, 10.1111/iwj.14632.38156706 PMC10961866

The above article, published online on 29 December 2023, in Wiley Online Library (http://onlinelibrary.wiley.com/), has been retracted by agreement between the journal Editor in Chief, Professor Keith Harding; and John Wiley & Sons Ltd. Following an investigation by the publisher, all parties have concluded that this article was accepted solely on the basis of a compromised peer review process. In addition, further investigation by the publisher found that the methodology lacked sufficient detail, that the discussion and conclusions were not supported by the cited references, and that the conclusions included contradictory statements to the article's stated outcomes. The editors have therefore decided to retract the article. The authors did not respond to our notice regarding the retraction.

**RETRACTION:** Y. Zhang , J. Li , and M. Liu , “Efficacy of Stem Cell Therapy for Diabetic Foot: Clinical Evidence From Meta‐Analyses,” International Wound Journal 21, no. 4 (2024): e14632, 10.1111/iwj.14632.38156706 PMC10961866

The above article, published online on 29 December 2023, in Wiley Online Library (http://onlinelibrary.wiley.com/), has been retracted by agreement between the journal Editor in Chief, Professor Keith Harding; and John Wiley & Sons Ltd. Following an investigation by the publisher, all parties have concluded that this article was accepted solely on the basis of a compromised peer review process. In addition, further investigation by the publisher found that the methodology lacked sufficient detail, that the discussion and conclusions were not supported by the cited references, and that the conclusions included contradictory statements to the article's stated outcomes. The editors have therefore decided to retract the article. The authors did not respond to our notice regarding the retraction.

